# Head in the game: the impact of cognitive abilities on performance of National Football League quarterbacks

**DOI:** 10.3389/fpsyg.2025.1540498

**Published:** 2025-01-29

**Authors:** R. Thomas Boone, Nicholas S. Zambrotta, Andrew M. Manocchio, James K. Bowman

**Affiliations:** ^1^Department of Psychology, University of Massachusetts, Dartmouth, North Dartmouth, MA, United States; ^2^Department of Clinical Psychology, Mercer University, Atlanta, GA, United States; ^3^Great Neck Public Schools, Great Neck, NY, United States

**Keywords:** American football, NFL draft, elite athlete performance, cognitive abilities, quarterbacks

## Abstract

American football is a multi-billion-dollar industry and source of social identity and national pride. Recruiting top level players is a priority for franchises, coaches, teams, and fans. Utilizing data obtained from 42 National League Football (NFL) quarterbacks, collected at their respective Combine experience, the current study adds to existing research demonstrating that cognitive abilities, as measured by the Athletic Intelligence Quotient (AIQ), namely Visual Spatial Processing, Reaction Time, and Decision Making, all increase the predictive accuracy beyond the role of draft pick at the Combine. Reaction Time; Visual Spatial Processing and Decision Making to a lesser, but notable degree; predicted NFL performance metrics such as Career Approximate Value, Quarterback Rating, passing and rushing yards per game, turnover worthy plays, and throwing accuracy. The role of cognitive abilities, particularly in the critical position of quarterback in American football, is discussed.

## Introduction

Athletic contests, from youth teams to college to the Olympics to professional sports franchises, are a large component of how people around the world spend their leisure time. Team loyalties are an important part of many individuals’ social identities, as evidenced by the amount of money committed to sports teams by spectators, franchises, and supportive functions. Sports ETA’s “State of the Industry Report” for 2023 encapsulates the magnitude of the industry within the United States alone with its report of an economic impact totaling $128 billion (Sports Events and Tourism Association, 2024). There are several notable sports leagues in the United States, with American football and the NFL as a primary focus. The end of the season culminates in the “Superbowl,” noted for some of the highest TV ratings of the year, huge advertising pushes, and celebrities from the music industry vying for a chance to highlight the half-time show. With such a commitment in terms of identity, time, and money, it is no small wonder that assembling the best team possible is the primary goal of any NFL organization, which includes recruiting athletes who are most likely to be successful at the professional level.

The NFL Scouting Combine allows NFL personnel to evaluate potential players on a variety of physical, mental, and medical criteria. The Combine has a history dating back to the 1970s, when teams began using interviews and medical exams to examine potential draft picks (NFL Scouting Combine, 2024). In the 1980s, the NFL Combine became centralized and moved to Indianapolis, where it remains today. Consequently, teams were able to devote more time to evaluating a prospect’s physical and mental ability. Even at this time, there was a strong emphasis on the mental and cognitive skills of potential draftees, with some teams prioritizing intelligence and character among their most important traits when evaluating players (Hickey, 1983). Beginning in the 1970s, the NFL utilized the Wonderlic Personnel Test as a standard measure of intellectual ability (Wonderlic and Hovland, 1939; Wonderlic, 2013). The traditional Wonderlic test includes 50 questions, is completed in 12 min, and measures general cognitive ability in the largely academic domains (e.g., mathematical problem-solving, vocabulary, and reasoning). Today, the standard assessment process includes screenings, tests, medical evaluations, physicals, and interviews of more than 300 invited players each year. Further, additional tests or evaluations (i.e., MRI or other diagnostic imaging) may be ordered or requested by teams.

Cognition and athletic performance are clearly linked, though the directionality and impact have not been fully explained. Activity in later life has been shown to predict greater cognitive health (Engeroff et al., 2018), and there is evidence that active participation in sport increases performance on cognitive tasks, connected down to the neurological level as evidence by increased P300 waves as measured by EEG (Aly et al., 2019; Aly et al., 2024). However, how these broader connections relate to elite athletic performance is unclear. Scores on the Wonderlic have not demonstrated strong predictive validity with respect to NFL player performance (Kuzmits and Adams, 2008; Lyons et al., 2009; Mann et al., 2007; Voss et al., 2010). Some researchers have argued that the test is culturally biased (Gill and Brajer, 2012). As a result, in 2022, the NFL announced that they would not only discontinue utilization of the Wonderlic, but would also discontinue administration of *any* psychological assessments at the Combine due to concerns of over-burdening players (O’Connell, 2022; Penn, 2022).

The NFL’s decision to drop psychological assessment at the Combine has decentralized testing. However, this decision has not slowed the development or use of new assessments, many of which were underway for over a decade given the lack of predictive validity for the Wonderlic. The NFL Player Assessment Test (NFL-PAT) (Siena Consulting, 2013) was developed in 2013 to measure learning styles, decision-making skills, core intellect, and the ability to respond to unexpected stimuli (Breer, 2013; Howard, 2013). Since inception, The NFL-PAT has been used annually by the NFL while it continues to be refined using feedback from teams and players (NFL, 2021). However, the PAT has suffered from a lack of peer-reviewed empirical research demonstrating relationships between test scores and game performance (Bowman et al., 2024). Other, more recently developed assessments, like APTUS and S2, also lack published empirical support for predictive validity in relation to on-field performance in the NFL.

The Athletic Intelligence Quotient (AIQ) (Bowman et al., 2020) measures sport-specific intelligence using a range of cognitive abilities (i.e., Decision Making, Reaction Time, Learning Efficiency, and Visual Spatial Processing) and was first utilized at the NFL Scouting Combine in 2012. Empirical support for the AIQ has been accumulating, with AIQ scores predicting performance metrics across three different sports, including unique predictive power beyond the usual metrics that go into recruitment as measured by draft pick. With regard to Major League Baseball (MLB), Visual Spatial Processing predicts Hitting statistics and Reaction Time predicts Pitching Outcomes, such as ERA (Bowman et al., 2021). In the NBA, Player Efficiency Rating (PER) and Effective Field Goal Percentage were predicted by Decision Making above and beyond the variables accounted for by Draft Pick (Hogan et al., 2023). In the NFL, Bowman et al. (2020) demonstrated that Reaction Time predicted Rushing Yards for running backs and Receiving Yards for wide receivers.

In addition to the global utility of the AIQ to predict broad outcomes, the AIQ has been successful in predicting more focused outcomes as well. In a recent study of NFL Offensive Linemen, both subscales of the Reaction Time index, which included a component focused on response accuracy, predicted the number of false starts per game, where players who were more impulsive were more likely to have a greater number of false starts in subsequent seasons (Bowman et al., 2024). In the analysis of MLB players, the interaction between Visual Spatial Processing and Reaction Time revealed a pattern suggesting that lower ERA was related to offsetting effects of faster reaction time or better visual spatial processing (Bowman et al., 2021).

The goal of the current study was to explore the role of global and focused effects of the cognitive abilities assessed by the AIQ on quarterbacks in the NFL. Intuitively, the role of the quarterback has a sizeable cognitive component. On the playing field, all offensive plays run through the quarterback, with each play typically having two or three alternatives to accomplish forward movement of the team. Ultimately, the quarterback is tasked with deciding which of these options to pursue at the start of play throughout the entire down. If the play is intended to be a pass play, there are likely two or three receivers who are designated as primary, secondary, or possibly tertiary receivers, one of whom is selected by the quarterback in real time as the play unfolds. While reduced in complexity, a series of competing choices also applies to the running game. To decide on the iteration that will lead to the greatest chance of success, the quarterback must consider the full range of the playbook (Learning Efficiency), map out where his players are moving (Visual Spatial Processing), assess the defensive formation (Visual Spatial Processing), and make a decision on whom to move the ball to (Decision Making), all while making sure that he is not tackled (Reaction Time).

Given the aforementioned cognitive demands, we predict that several AIQ factors will predict performance statistics of quarterbacks in the NFL. Specifically, we predict that Reaction Time will be related to most performance metrics. We also predict that both Decision Making and Visual Spatial Processing, and possibly the interaction between the two abilities, will impact passing statistics. To be consistent with previous work, we included Learning Efficiency in our initial analyses, but given the lack of findings involving this subscale to date, we offer no hypotheses involving it. We used the AIQ measures obtained at the time of recruitment, prior to quarterbacks’ involvement across one or more seasons in the NFL, to evaluate the predictive power of AIQ factors on performance in the NFL. We specifically focused on summative performance statistics such as Career Approximate Value (CAV) and QB rating; individual performance metrics such as games started, passing yards, and rushing yards; and Professional Football Focus (PFF) metrics, including Big Time Throw rate, Turnover Worthy Plays, and Throwing Accuracy.

## Methods

### Participants

From 2014 to 2020, the AIQ was administered to 46 quarterbacks who had declared for the NFL draft prior to their advancement to the NFL. To ensure that the statistics were mostly comparable across players, we excluded data from any player who did not play more than three games in total, reducing our sample size to 42. While the smaller sample size does impact power, this is one of the larger samples available for this focused population of interest.

### Ethical considerations

Participation in the current study was voluntary and further included an informed consent process that was directed by the testing proctor prior to the draftee completing the AIQ. The informed consent process was conducted in line with procedures as directed by NFL draft evaluation processes. Finally, all data employed in the current study was de-identified in order to preserve anonymity of participant data. The proposed secondary data analysis was approved by the UMass Dartmouth IRB in July of 2024.

### Instruments

#### Athletic intelligence quotient (AIQ)

The Athletic Intelligence Quotient is a measure designed to assess cognitive abilities that are particularly relevant for elite athletes (Bowman et al., 2020; Bowman et al., 2021; Bowman et al., 2024; Hogan et al., 2023). The measure aims to encapsulate domains of cognition in comprehensive fashion by delivering 10 subtests in a specific, consistent order to all participants via a software program on a tablet. Estimated time for full administration typically ranges from 35 to 38 min.

Bowman et al. (2020) describes the development of the AIQ, including details on how it was normed using professional athletes and how the *a priori* model was supported by a CFA affirming the four factor structure. Bowman et al., 2020 details how the 10 subtests of the AIQ map on to the four domains of cognitive ability including Visual Spatial Processing, Reaction Time, Decision Making (listed as Processing Speed in the Bowman et al., 2020 article), and Learning Efficiency. We investigate the relationship of these four areas of cognition with a range of metrics that are subsequently described (e.g., PFF Statistics, QBR). Following are brief descriptions of the four major subscales of the AIQ and the name(s) of the associated subtests.

##### Visual spatial processing

Quarterbacks are tasked with visualizing the playing field, processing possible outcomes prior to the snap of the game ball, adapting as the play develops, and optimizing the outcome of the play based on such developments. Overall, as measured by the Visual Spatial Processing subscale, this position requires skillsets in manipulation of stimuli, visual retention, navigation, and spatial awareness. This subscale is a composite of the Visualization subtest, the Spatial Relations subtest, the Visual Memory subtest, and the Spatial Scanning subtest.

##### Reaction time

On the playing field, quarterbacks process various sets of stimuli that have implications for the development of a play. However, regardless of stimuli, the speed at which the quarterback responds to a target stimulus can affect opportunities within a play (e.g., tucking the ball when an inevitable, oncoming sack is detected). Further, the quarterback is responsible for filtering the most relevant stimuli that are of primary importance, requiring the inhibition of reactions that would negatively impact a play’s optimal outcomes. This subscale is a composite of the Simple Reaction Time and Choice Reaction Time subtests.

##### Decision making

Much like the Reaction Time metric, Decision Making pertains to understanding how a quarterback may process stimuli that are relevant to the target play. Decision Making is differentiated in that it assesses the quarterback’s ability to scan and identify relevant stimuli to which they may respond efficiently and accurately thereafter. This maps onto abilities in scanning and identification of pre-snap reads as well as other in-game decisions. Succinctly, quarterbacks are positioned to make decisions pre-and post-snap that have consequences for the remainder of the play, demanding that both speed and accuracy accompany such decisions. This subscale is a composite of two separate measures of perceptual speed.

##### Learning efficiency

The quarterback position requires players to have a comprehensive understanding of the playbook, a capacity to retrieve such information, as well as flexibility in the learning of the playbook. Learning Efficiency not only encapsulates how quickly the quarterback is able to do so, but also indicates how readily the individual is capable of recalling this information in-game. Additionally, a quarterback must learn in real-time during games and flexibly apply learned information to novel circumstances (e.g., adapting to results from the first quarter of play). This subscale is a composite of two different measures of associative memory.

#### Pro football reference summative metrics

##### Career approximate value

Estimates the value that a positional player contributes to their teams over the course of their career. The metric aims to represent a player’s production with a single number by calculating the player’s contributions to the position of interest within their team’s overall performance.

##### Quarterback rating

This metric demonstrates a quarterback’s overall performance by incorporating data that includes, but is not limited to, passing yards, rushing yards, touchdowns, turnovers, etc. (Katz and Burke, 2016). Much like the CAV metric, we see utility in considering a single number that may represent the skill and/or performances of a quarterback in a standardized manner.

#### Pro football reference quarterback specific statistics

These official game statistics are tallied and updated after each game. The full number of statistics available is quite large, but in the interest of parsimony we elected to settle on a few of the more prominent metrics. To evaluate whether coaches felt confidence in their quarterback, we selected the number of games players started. To assess the running game, we obtained the number of rushing yards per game. To assess skill in the passing game, we obtained the number of passing yards per game.

#### Pro football focus (PFF) statistics

Pro Football Focus (PFF), originally founded by Neil Hornsby in 2006 and currently owned and managed by Chris Collinsworth, provides relevant statistics by documenting game actions for all players within each game in the NFL (Monson and Palazzolo, 2018). For the current study that is interested in investigating quarterback performance and its correlates with AIQ-measured cognitive abilities, three metrics were of particular interest: Big Time Throw rate (BTT), Turnover Worthy Play (TWP), and Throwing Accuracy (TA).

##### Big time throw rate

PFF grades BTT according to balls thrown at a large distance, which would inherently mean they are more susceptible to reduced accuracy. The metric accounts for such balls that are nonetheless well-placed and grant a good opportunity for the target receiver to complete a catch.

##### Turnover worthy play

Quarterbacks are measured on this metric by PFF by accounting for (1) passes that are poorly placed, which increases risk for an interception, and (2) placing the ball at risk of being fumbled.

##### Throwing accuracy

This metric accounts for ball placement that maximizes likelihood of completion to a target receiver. This metric also includes consideration of ball placement that may maximize opportunity for the receiver to find yards-after-the-catch.

### Procedure

The AIQ was administered on behalf of specific teams as part of the NFL pre-draft process by AIM, LLC from the years 2014 to present. Prior to the start of testing, consent was obtained after draftees were informed of important details, including the purpose of the assessment and who would have access to the results. They were also told they could discontinue the assessment at any time, and they could receive feedback about their performance afterwards.

Participants were then directed by test administrators to a quiet space with no distractions. All participants were delivered a standardized introduction to the AIQ assessment, including a brief description of the AIQ, information regarding presentation of both visual and audio instructions for which they would be provided earphones, as well as a prompt to give their best effort. Once the instructions were delivered and understood, the test administrator initialized the AIQ software. Of note, trained test administrators proctored the evaluation space to ensure that test instructions were properly understood by participants and delivered with fidelity.

AIM, LLC tracked the various performance metrics identified above and matched them to AIQ scores of the draftees who are now NFL quarterbacks, covering the seasons from 2018 to 2021. The company then removed the identifying information from the dataset and provided the de-identified data to our research team on 07/28/2024. All analyses were performed using IBM SPSS 29.0. For the purposes of protecting the private cognitive performance data of the quarterbacks, we have avoided presenting individual level data in our results.

## Results

### Data analytic strategy

The current data set is quite unique and understandably small in size. As such we wanted to test very specific hypotheses, specifically the relationship between the four AIQ subscales after controlling for Draft Pick. To fully control for Draft Pick first, we opted for a regression approach. All initial analyses included all these variables, and then for the purposes of parsimony and to remove non-predictive overlap among the four AIQ variables, we reported the trimmed models, only saving the variables that were significant in the initial analysis. Thus, the full model was run first, variables that were significant in the full model were retained for the trimmed model, balancing the modest collinearity of the AIQ subscales while avoiding a spurious patten based on *p*-values. Based upon previous findings in MLB and the NBA, we also planned for some interaction hypotheses for our later variables if we found main effects for Visual Spatial Processing.

### Draft pick and games started per year

One of our goals is to demonstrate the utility of using the AIQ above and beyond the impact of previous assessments of player ability. To that end, these previous assessments can be readily captured by Draft Pick. Draft Pick represents the summative desirability of the player by the collective scouts, coaches, and front office personnel of the NFL at the time of selection. Games Started Per Year reflects the ongoing decision of coaches to start a quarterback ahead of other players on their roster. To answer the question of whether the four subtests of the AIQ were related to either Draft Pick or Games Played, we ran the zero-order correlations, reported in Table 1. Neither of the two measures, Draft Pick or Games Played, were directly related to AIQ subtests.

**Table 1:** Zero-order correlations for games played, pick, and AIQ factors (*N* = 42).

	Visual spatial processing	Reaction time	Decision making	Learning efficiency
Pick	0.18	0.13	0.26^†^	0.11
Games started per year	−0.09	0.14	−0.20	−0.06

^†^*p* < 0.10, *p < 0.05, **p < 0.01, ***p < 0.001.

However, it would be very surprising if Draft Pick was not related to Games Started Per Year and, in the spirit of exploring the contribution of the AIQ above in and beyond the effect of Draft Pick, we ran a hierarchical multiple regression with Games Started per Year as the dependent variable and the four AIQ subscales as predictor variables, after controlling for draft pick. The overall model was significant, *F*(5,36) = 13.5, *p* < 0.001, However, given the modest overlap between the AIQ measures, we elected to present the trimmed model; here and throughout the remaining regressions presented in this paper. As shown in Table 2, after controlling for the effect of Draft Pick, the AIQ Reaction Time subscale predicted an additional 6% of the variance of how many games a quarterback started.

**Table 2:** Hierarchical regression of games started per year as a function of draft pick and AIQ factors (*N* = 42).

Step and predictor variables	*R^2^*	∆*R^2^*	*sr^2^*	*β*	SEB
Games started per year					
Step 1	0.53^***^	0.53 ^***^			
Draft pick			−0.73**	−0.73	0.009
Step 2	0.60^***^	0.07^*^			
Reaction time			0.26^*^	0.26	0.055

^†^*p* < 0.10, *p < 0.05, **p < 0.01, ***p < 0.001.

### Pro football reference summative statistics

To evaluate the effect of the four AIQ subscales on overall performance, we obtained each player’s Career Approximate Value per year (CAV) and Quarterback Rating (QBR) from profootballreference.com. Using the same hierarchical regression model with Draft Pick entered first, followed by the four AIQ Subscales trimmed as needed separate regressions were performed for each dependent variable. Table 3 shows the descriptive statistics and zero-order correlations for both CAV and QBR. Reaction Time showed a marginal zero-order effect on CAV. The two regression analyses showed a slightly clearer picture. Our initial model focusing on CAV was significant, *F*(5,36) = 9.37, *p* < 0.001. As shown in Table 4, the trimmed model for the CAV regression remained significant, *F*(2,39) = 9.04, *p* < 0.001. Both Draft Pick and Reaction Time were significant unique predictors and Reaction Time explained for an additional 13% of the variance after accounting for the effect of Draft Pick. Higher scores on the reaction time measure, which were standardized so that higher scores meant faster and more accurate reaction times, predicted higher CAV values, even after accounting for Draft Pick.

**Table 3:** Descriptive statistics and zero-order correlations for CAV per year and QBR and AIQ factors (*N* = 42).

	*M*	*SD*	Visual spatial processing	Reaction time	Decision making	Learning efficiency
CAV	5.64	5.10	−0.10	0.26†	−0.11	−0.08
QBR	79.01	18.12	0.15	0.20	−0.13	−0.01

^†^*p* < 0.10, *p < 0.05, **p < 0.01, ***p < 0.001.

**Table 4:** Hierarchical regression of CAV as a function of draft pick and AIQ factors (*N* = 42).

Step and predictor variables	*R^2^*	∆*R^2^*	*sr^2^*	β	SEB
CAV					
Step 1	0.44^***^	0.44 ^***^			
Draft pick			−0.66^***^	−0.66	0.008
Step 2	0.56^***^	0.13^**^			
Reaction time			0.36^**^	0.36	0.051

^†^*p* < 0.10, *p < 0.05, **p < 0.01, ***p < 0.001.

The regression for QBR showed a similar pattern. As shown in Table 5, the trimmed model for the CAV regression was significant, *F*(2,39) = 11.2, *p* < 0.001. Both Draft Pick and Reaction Time were significant unique predictors and Reaction Time explained for an additional 8% of the variance after accounting for the effect of Draft Pick. Better reaction time scores predicted higher QBR values.

**Table 5:** Hierarchical regression of QBR as a function of draft pick and AIQ factors (*N* = 42).

Step and predictor variables	*R^2^*	∆*R^2^*	*sr^2^*	β	SEB
QBR					
Step 1	0.32^***^	0.32 ^***^			
Draft pick			−0.57^***^	−0.57	0.033
Step 2	0.40^***^	0.08^*^			
Reaction time			0.28^*^	0.28	0.23

^†^*p* < 0.10, *p < 0.05, **p < 0.01, ***p < 0.001.

### Pro football reference quarterback specific statistics

To evaluate the effect of the four AIQ subscales on yards gained, both passing and rushing, we calculated each player’s average passing and rushing yards per game by dividing the number of career passing/rushing yards accumulated by the number of games played. Using the same hierarchical regression model with Draft Pick entered first, followed by the four AIQ Subscales, separate regressions were performed for both variables. Table 6 shows the descriptive statistics and zero-order correlations. Reaction Time reveals a marginal relationship with yards rushing.

**Table 6:** Descriptive statistics and zero-order correlations for passing yards per game and AIQ factors (*N* = 42).

	*M*	*D*	Visual spatial processing	Reaction time	Decision making	Learning efficiency
Passing yards per game	165.72	78.54	0.16	0.07	−0.08	0.10
Rushing yards per game	15.68	13.7	−0.16	0.24	−0.05	−0.11

^†^*p* < 0.10, *p < 0.05, **p < 0.01, ***p < 0.001.

Again, the hierarchical regression revealed a more nuanced picture. As shown in Table 7, the trimmed model for the Passing Yards regression was significant, *F*(2,39) = 16.5, *p* < 0.001. Both Draft Pick and Visual Spatial Processing were significant unique predictors and Visual Spatial Processing explained for an additional 7% of the variance after accounting for the effect of Draft Pick. Better Visual Spatial Ability predicted more passing yards.

**Table 7:** Hierarchical Regression of Passing Yards per Game as a Function of Draft Pick and AIQ Factors (*N* = 42).

Step and predictor variables	*R^2^*	∆*R^2^*	*sr^2^*	β	SEB
Passing yards per game					
Step 1	0.39^***^	0.39 ^***^			
Draft pick			−0.62^***^	−0.62	0.129
Step 2	0.46^***^	0.07^*^			
Visual spatial processing			0.27^*^	0.27	1.14

^†^*p* < 0.10, *p < 0.05, **p < 0.01, ***p < 0.001.

AIQ measures also added additional predictive power to Rushing Yards per game. As shown in Table 8, the trimmed model for the Passing Yards regression was significant, *F*(2,39) = 6.0, *p* = 0.005. Both Draft Pick and Reaction Time were significant unique predictors and Reaction Time explained for an additional 9% of the variance after accounting for the effect of Draft Pick. Better reaction time outcomes predicted more rushing yards.

**Table 8:** Hierarchical regression of rushing yards per game as a function of draft pick and AIQ factors (*N* = 42).

Step and predictor variables	*R^2^*	∆*R^2^*	*sr^2^*	β	SEB
Rushing yards per game					
Step 1	0.15^*^	0.15^*^			
Draft pick			−0.39^*^	−0.39	0.025
Step 2	0.24^**^	0.09^*^			
Reaction time			0.30^*^	0.30	0.176

^†^*p* < 0.10, *p < 0.05, **p < 0.01, ***p < 0.001.

### PFF statistics

The PFF statistics offered an additional opportunity for analysis since we were able to obtain data on a yearly basis, affording the opportunity to use a mixed model data-analytic strategy where year was a level one variable nested within individual player (level 2). While we were not interested in variations per year, the ability to more accurately parse the variance and reduce the within player variance greatly enhanced the power of these analyses. Further, this type of analysis is flexible enough to accommodate a situation where there are a differing number of cases of a level 1 variable. For example, we had some cases with 5 years of data points and other with merely one year of data.

We adopted a global strategy in our approach to analyzing each of the three targeted statistics. First, we again examined all three dependent variables for outliers. BTT did not have any significant outliers, but TWP and Throwing Accuracy did. As suggested by Tabachnick and Fidell (2013), we winsorized the data by giving any value more than three standard deviations above the mean a value of 1 unit greater than that cutoff value. This strategy preserves the large value relative to the other data, but does not allow the extreme value to unduly impact subsequent analyses. Additionally, since the mixed model analytic strategy relies on a regression model, we also did a z-transform on all the predictor variables used on level 2 of the analysis to readily center the data in anticipation of possible interaction effects.

#### Big time throw rate (BTT)

We entered each player’s BTT value per year into a Mixed Model Analysis where year was a level one variable nested within level 2, the individual. Our initial model included all normed values of draft pick and the four AIQ factors. However, in running that analysis, none of the AIQ subscales significantly predicted BTT. As shown in Table 9, only draft pick was significantly related to BTT with lower draft pick numbers associated with higher BTT.

**Table 9:** Mixed model analysis of BTT as a function of draft pick and AIQ factors.

Step and predictor variables	*b*	SEB	95% CI	
			LB	UB
Big time throw				
Draft pick	−0.014*	0.005	−1.82	−0.37

^†^*p* < 0.10, *p < 0.05, **p < 0.01, ***p < 0.001.

#### Turnover worthy play (TWP)

**Figure 1:**
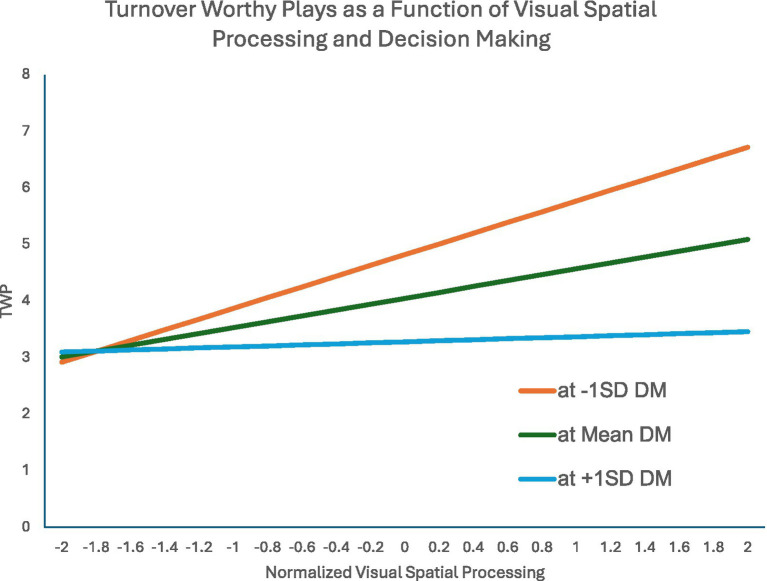
TWP=Turnover Worthy Plays; DM = Normalized Decision Making; Lines are predicted regression lines of Visual Spatial Processing on Turnover Worthy Plays at three diBerent levels of Decision Making (minus one standard deviation of Decision Making, mean Decision Making, and plus one standard deviation of Decision Making).

We entered the winsorized TWP variable in a parallel analysis to what was done with BTT, with the normalized draft pick, the AIQ variables, and the predicted interaction between Visual Spatial Processing and Decision Making as predictor variables. Reaction Time was not significant, so we re-ran the analysis with that variable removed. As shown in Table 10, draft pick was not significantly related to TWP, but Visual Spatial Processing, Decision Making, and the interaction term were significant. As is the case with significant interaction terms, interpretations should start there. Figure 1 illustrates the effect: When Decision Making scores are higher, Visual Spatial Processing appears unrelated to Turnover Worthy Plays. When Decision Making skills are average or below average, higher Visual Spatial Processing is associated with a higher number of Turnover Worthy Plays.

**Table 10:** Mixed model analysis of TWP as a function of draft pick and AIQ factors.

Step and predictor variables	*b*	SEB	95% CI	
			LB	UB
Turnover worthy play				
Pick	0.18	0.23	−0.30	0.65
Visual spatial processing	0.52*	0.24	0.03	1.01
Decision making	−0.77**	0.32	−1.26	−0.29
VSP X DM	−0.434*	0.22	−0.86	−0.004

^†^*p* < 0.10, *p < 0.05, **p < 0.01, ***p < 0.001.

#### Throwing accuracy

**Figure 2:**
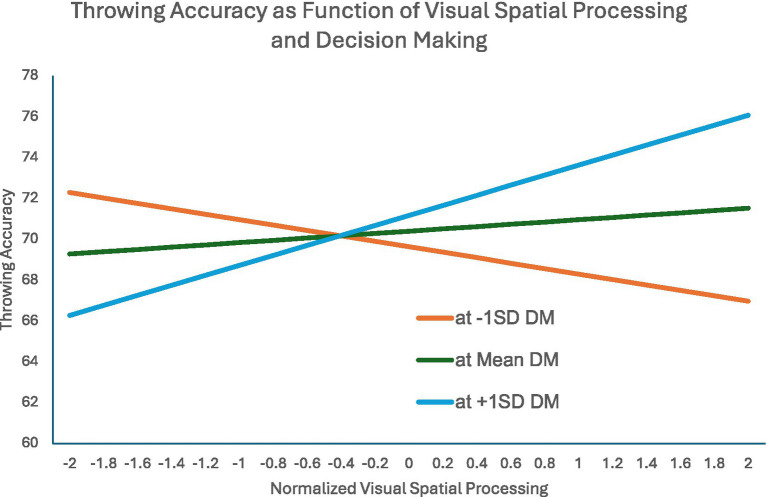
DM = Normalized Decision Making; Lines are predicted regression lines of Visual Spatial Processing on Throwing Accuracy at three diBerent levels of Decision Making (minus one standard deviation of Decision Making, mean Decision Making, and plus one standard deviation of Decision Making).

We entered the winsorized Throwing Accuracy variable in a parallel analysis to what was done with BTT and TWP, the normalized draft pick, the AIQ variables, and the predicted interaction between Visual Spatial Processing and Decision Making as predictor variables. As shown in Table 11, main effects for both Draft Pick and Reaction Time were significant: Lower Draft Pick numbers and better Reaction Time scores were associated with greater Throwing Accuracy. While the main effects of Visual Spatial and Decision Making scores were not significant as main effects, the interaction term was. Figure 2 depicts that interaction effect. For average Decision Making scores, Visual Spatial Processing had a little to a moderate effect on Throwing Accuracy. However, for lower Decision Making scores, Visual Spatial Processing was negatively related to Throwing Accuracy and with higher Decision Making scores, Visual Spatial Processing was positively related to Throwing Accuracy. It is worth noting that for quarterbacks with high Visual Spatial Processing and high Decision Making scores, their Throwing Accuracy was 6% greater than quarterbacks with average values on these measures.

**Table 11:** Mixed model analysis of accuracy rate as a function of draft pick and AIQ factors.

Step and predictor variables	*b*	SEB	95% CI	
			LB	UB
Accuracy rate				
Draft pick	−3.03**	0.84	−4.72	−0.13
Reaction time	1.89*	0.86	0.12	3.67
Visual spatial processing	0.56	0.86	−1.20	2.31
Decision making	0.77	0.89	−1.04	2.59
VSP X DM	1.85*	0.77	0.31	3.40

^†^*p* < 0.10, *p < 0.05, **p < 0.01, ***p < 0.001.

## Discussion

The goal of the current study was to map out the potential relationship between a cognitive performance measure (AIQ) and performance metrics of quarterbacks in the NFL. Specifically, the AIQ is a tool assessing four cognitive domains that we believe may be related to certain aspects of quarterback performance. Quarterbacks play a distinct role in the game of American football, in that they are the central nexus of every offensive play and their performance is based as much on the decisions they make as much as it is on their innate athleticism. Within such a configuration, we expected to find that cognitive performance measures would significantly predict the statistics relating to quarterback performance across an array of outcome metrics. Given that our hope was to demonstrate that cognitive performance measures offered an additional and unique contribution beyond what is typically included in the recruitment process, we accounted for draft pick first, and then looked for the effect of the cognitive performance measures above and beyond the initial predictions of draft pick.

Overall, our hypotheses were supported; there were several quarterback performance metrics that were predicted by AIQ cognitive factors. First, it is important to note that draft pick was a significant predictor of all but one metric we evaluated. This finding is not surprising, we would hope that all the processes put into recruitment would have yielded an effective estimate of any player’s future performance. Although relatively new to consideration in the recruitment process, some combinations of our cognitive performance indices added additional predictive power to six of the seven quarterback performance metrics.

We utilized two sources of quarterback performance metrics, pro football reference statistics and PFF statistics. The pro football reference statistics included Career Approximate Value and the QBR indices, both of which were significantly predicted by the AIQ Reaction Time subscale, after controlling for draft pick. Quarterbacks with better performance on this task had higher CAV and QBR indices. It is not surprising that athletes with quicker reaction times do better, which is notably differentiated from a physical measure like sprinting speed which is part of the draft pick formulation. Rather, Reaction Time represents a cognitive ability which surely impacts the speed of cognitive appraisals quarterbacks have to make in any given play. The pro football reference statistics also included passing yards and rushing yards and here we again obtained evidence of the contribution of cognitive factors, particularly, visual spatial skills predicted passing yards and reaction time predicted rushing yards. Quarterbacks with greater respective skills demonstrated a greater ability to move the ball down the field via passing and rushing, above and beyond the assessments that coaches and franchises made when evaluating the the players’ position in the draft.

The PFF statistics had an additional advantage in that it often made available years of data on player performance, leading to a more powerful mixed model analysis. While Big Time Throw rate was not impacted by any of our cognitive measures, both Turnover Worthy Plays and Throwing Accuracy showed an effect for the interaction between Visual Spatial Ability and Decision Making. In the case of Turnover Worthy Plays, the interaction seems to suggest that higher visual spatial skill is not distinctly an advantage, but, when coupled with poor decision making, can lead to a greater number of negative outcomes – more Turnover Worthy Plays. In the case of Throwing Accuracy, the pattern suggests that good visual spatial skills paired with good decision making is a distinct advantage, but good visual spatial skills paired with poor decision making can lead the quarterback astray. Throwing Accuracy was also predicted by reaction time, presumably with an advantage for getting the ball to the receiver as soon as he is open. Collectively, these findings suggest that higher visual spatial skill allows the quarterback to see the options more clearly, but seeing the option is not enough, and the quarterback must still make the correct decision. When placed in the context of the need to execute a play and an overall ethos toward action, it is not surprising to see a default to commit when the information, good or not, is presented.

In summary, Reaction Time had a consistent effect across most of the performance metrics we assessed, suggesting that it is a broad advantaged cognitive skill in predicting elite athletic performance. Visual Spatial Processing and Decision Making were more narrowly focused, primarily in the area of the passing statistics. Such focus should not be surprising given the demands of making a successful completion. It is also worth noting that, consistent with our other published work, there was no effect of Learning Efficiency on any of the metrics we assessed.

### Limitations and directions for further research

While we have shown that the AIQ has utility in predicting quarterback performance, some discussion of the limitations of the current study should be addressed. As with many studies, particularly ones with a specialized target population, our sample size is small and there is a risk of a sampling bias. Our subset of quarterbacks does appear to fall within the general range of all quarterbacks with regard to draft pick and performance metrics, and our findings in related research suggest that this is likely to be a stable pattern. Additionally, while the hope is that unmeasured variables would be randomly distributed as error, clearly we have left a number of other likely predictive factors out of our model, including coaching variables, other team members, including offensive line quality, and team-specific offensive scheme. Our model focuses on individual level data, but multi-level analyses and a comprehensive data set theoretically allows the possibility of combining individual level variables with team level variables for a more fully detailed model.

Separately, there are most likely important psychological facets of successful quarterback play that are not captured with cognitive evaluations. While more work might be required to adequately measure such constructs in this population, examples of such facets might include arousal regulation, resilience, and emotional functioning. Thus, the AIQ is not, nor should it be, considered an exhaustive measure of the psychological skills related to successful quarterback play in the NFL.

Additionally, while we used draft pick status as a proxy for summative desirability of the player by the collective scouts, coaches, and front office personnel of the NFL, it is important to note that being highly drafted is not a guarantee of success on the field. Many quarterbacks taken high in the draft underperform. Further, it is not uncommon for quarterbacks taken in late rounds of the draft to have successful careers in the NFL. Another limitation is using games started per year as a proxy metric of ability. While it is generally true that better players will play sooner, it may also be true that since teams have invested more resources into top draft picks, teams may be incentivized to sideline such players to augment their development behind a veteran or lower draft capital player.

Nevertheless, the AIQ continues to demonstrate predictive validity regarding performance in the NFL. Future research related to the AIQ and NFL performance could include closer examination of the specific narrow cognitive abilities that impact quarterback performance (rather than simply the 4 broad cognitive abilities). Such an examination could be particularly helpful in the draft process when teams are often searching for players that play a particular position or set of positions. Additionally, future research might include other professional sports (e.g., ice-hockey, soccer/football, Australian rules football, etc.), or elite Olympic athletes. Future work may also include the AIQ’s ability to predict amateur or collegiate athlete performance. Indeed, given the relative restricted range likely to occur among high-level professional athletes, the impact of these cognitive factors may be greater among amateur or collegiate athletes.

There is considerable interest in the field as to whether athletes can be trained to improve their perceptual or cognitive abilities with transfer to sports performance (Fransen, 2024; Harris et al., 2018). While there have been a number of tests and interventions launched, the overall utility seems to be underwhelming (Harris et al., 2018). Some critiques focus on the relative rarity of “far transfer,” (Fransen, 2024). While others suggest that the modular approach, particularly when divorced from the ecological environment of skill deployment, is likely to fail (Renshaw et al., 2019). Our research takes advantage of the inherent variability on these measures across athletes and demonstrates the utility of the cognitive measures on specific sports performance. Our research does not address whether such skills can be trained, but the specificity of our findings and the direct connection to sports performance outcomes suggest a future avenue for researchers hoping to develop a successful training platform, assuming that the correct theoretical framework is applied (Hadlow et al., 2018). Such a training platform would also be enhanced by evaluating some of the neurological processes as suggested by Aly et al. (2024). Should future research address the opportunity to train such skills. Scouts and coaching staff would ideally be able to address growth areas identified via the AIQ via coaching to improve an athlete’s on-field performance.

In sum, our current findings contribute to the overall corpus of research (i.e., Bowman et al., 2020; Bowman et al., 2021; Hogan et al., 2023; Bowman et al., 2024) demonstrating the importance of cognitive ability in professional sports. Certainly, physical abilities contribute heavily to success, but the cognitive factors should not be ignored.

## Data Availability

The raw data supporting the conclusions of this article will be made available by the authors, without undue reservation.
